# Early Impacts of the COVID-19 Pandemic on the United States Construction Industry

**DOI:** 10.3390/ijerph18041559

**Published:** 2021-02-06

**Authors:** Abdullah Alsharef, Siddharth Banerjee, S M Jamil Uddin, Alex Albert, Edward Jaselskis

**Affiliations:** 1 Department of Civil, Construction, and Environmental Engineering, North Carolina State University, 2501 Stinson Dr., Raleigh, NC 27607, USA; sbaner22@ncsu.edu (S.B.); suddin@ncsu.edu (S.M.J.U.); alex_albert@ncsu.edu (A.A.); ejjasels@ncsu.edu (E.J.); 2 Civil Engineering Department, College of Engineering, King Saud University, P.O. Box 22452, Riyadh 11451, Saudi Arabia

**Keywords:** COVID-19, COVID-19 risk, construction safety, occupational safety, lessons learned, mitigation strategies, construction delays, construction productivity, worker safety, safety risk

## Abstract

The COVID-19 pandemic has been the largest global health crisis in decades. Apart from the unprecedented number of deaths and hospitalizations, the pandemic has resulted in economic slowdowns, widespread business disruptions, and significant hardships. This study focused on investigating the early impacts of the COVID-19 pandemic on the U.S. construction industry since the declaration of the national emergency on 13 March 2020. The study objectives were achieved through 34 telephone interviews with project managers, engineers, designers, and superintendents that represented different states and distinct industry sectors in the United States (U.S.). The interviewees offered information on their experience with the pandemic, including the general and adverse effects experienced, new opportunities created, and risk management efforts being undertaken. The reported adverse effects included significant delays on projects, inability to secure materials on time, reduction in productivity rates, material price escalations, and others. The new opportunities that were created included projects involving the fast-track construction of medical facilities, construction of residential buildings, transportation-related work, and opportunities to recruit skilled workers. The risk management measures that were widely adopted included measures to enhance safety and reduce other project risks. The safety measures adopted included requiring employees to wear cloth face masks, adoption of social distancing protocols, staggering of construction operations, offering COVID-19-related training, administering temperature checks prior to entry into the workplace, and others. Measures to manage other project risks included the formation of a task force team to review the evolving pandemic and offer recommendations, advocating that construction businesses be deemed essential to combat delays and taking advantage of government relief programs. The study findings will be useful to industry stakeholders interested in understanding the early impacts of the pandemic on the construction industry. Industry stakeholders may also build upon the reported findings and establish best practices for continued safe and productive operations.

## 1. Introduction and Study Motivation

The coronavirus disease also known as COVID-19 is caused by the virus named severe acute respiratory syndrome coronavirus 2 (i.e., SARS-CoV-2) [1]. The virus is confirmed as being transmitted from human to human and results in symptoms including fever, dry cough, fatigue, and shortness of breath [2]. Since the first cases were reported by the World Health Organization (WHO) on 31 December 2019, the virus has spread to over 200 nations [1]. The WHO declared the crisis as first being a public health emergency of international concern on 30 January 2020 [3]. Later, the crisis was declared as being a global health pandemic on 11 March 2020 [4].

**Figure 1:**
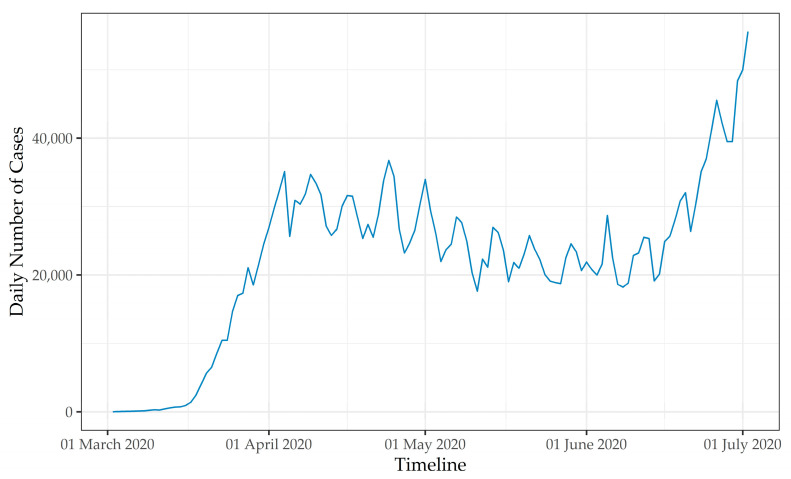
Daily number of confirmed COVID-19 cases in the U.S. between March and July 2020 (i.e., early impacts) as reported by the COVID Tracking Project [6].

Given the rapid spread in the U.S. following the first detected case in January, a national emergency was declared on 13 March 2020 [5]. Since then, the number of confirmed COVID-19 cases in the U.S. has continued to increase rapidly, as can be seen in Figure 1 [6]. As of 3 January 2020, over 20 million confirmed cases and more than 350,000 deaths had been linked with the COVID-19 pandemic in the U.S. [6]. Not surprisingly, the COVID-19 pandemic was identified as the leading cause of death in the U.S. in 2020 [7].

Apart from the widespread health crisis, the COVID-19 pandemic has resulted in a nationwide economic downturn. In fact, the National Bureau of Economic Research (NBER) announced that the U.S. entered a recession phase in February—and called it the COVID-19 recession [8,9]. Because of the economic downturn, the U.S. has experienced record-high unemployment rates. More specifically, from an unemployment rate of about 3.8% in February 2020, the unemployment rate peaked at roughly 14.7% in April 2020 [10]. This corresponds to over 23 million individuals in the U.S. being unemployed—far exceeding the numbers experienced at any time during the Great Recession (i.e., 2007 to 2009) [10]. The high unemployment rates resulted from the massive shrinkage in demand that has devastated industries including airlines, restaurants, manufacturing, and retail [11,12,13]. These unemployment rates have resulted in much financial distress among citizens; particularly among lower-income individuals and those that were unable to continue work [14].

**Figure 2:**
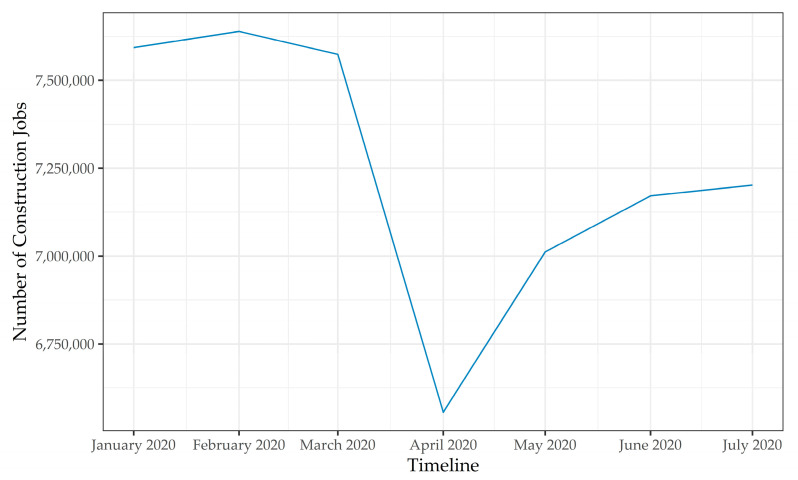
Construction industry employment data between January and July 2020 (i.e., early impacts) as reported by the Bureau of Labor Statistics (BLS) [15].

Like the other industries, the construction industry has also been impacted by the pandemic in a number of ways. For example, like other industries, the number of construction jobs available reduced following the pandemic onset—with the lower number of jobs reported in April 2020, as shown in Figure 2 [15]. These job losses are partly attributable to interruptions in work following work-related restrictions that were imposed to curb the virus spread, shortage in personal protective equipment (PPE) as it was prioritized for healthcare workers, and widespread market uncertainty. In addition, several construction projects were delayed and suspended; particularly in the oil and gas sector (e.g., West Loop Gas Pipeline, Liberty Pipeline, etc.), where the demand for oil dipped following travel restrictions [16,17].

However, most importantly, a significant number of construction workers reportedly tested positive for COVID-19. In fact, a recent investigation from Los Angeles concluded that construction workers were reporting the highest number of positive cases compared to workers in other industries, including transportation, healthcare, and manufacturing [18]. Likewise, another study found evidence that construction workers are roughly five times more likely to be hospitalized as a result of COVID-19 than workers in other industries [19]. Several other state public departments have also highlighted the risk of COVID-19 infections, particularly among the construction workforce [20,21,22].

Although some preliminary surveillance data on the impacts of the COVID-19 pandemic in the context of the construction industry exist, there is much that remains unknown. Insights from industry stakeholders are particularly lacking in the broader literature. Accordingly, the reported effort focused on gathering information on the effect of the COVID-19 pandemic from the perspective of the construction workforce. The effort also focused on identifying new opportunities that may have been created and efforts that were undertaken to manage the challenges associated with the pandemic.

The findings are expected to be useful as the industry continues to combat the pandemic and grapple with preserving safety and maintaining productivity. The findings can also serve as a resource for the future if the industry encounters similar epidemics, pandemics, or emergencies.

## 2. Research Methods

**Figure 3:**
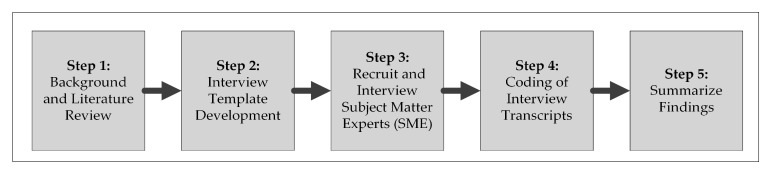
Adopted research process.

To accomplish the research objectives, the research methods, as presented in Figure 3, were adopted. As can be seen, following a review of the limited and relevant research, a semi-structured interview template was developed. As per the template, after gathering information on the participant’s background (i.e., professional role, workplace location, experience in no. of years, etc.), the responses to the following questions were targeted for solicitation:

- How has the COVID-19 pandemic affected the construction industry and the project(s) you are involved in (i.e., general and adverse impacts)?
- Have there been new opportunities for the construction industry as a result of the COVID-19 pandemic? If so, what are they?
- What efforts have been undertaken to manage the challenges associated with the COVID-19 pandemic in the context of the construction industry? Are there any related challenges that are being experienced?

As can be seen, the targeted questions covered three specific thematic areas that captured the impact of the pandemic on the industry. After the interview template was finalized, prospective subject matter experts (SMEs) were identified through professional organizations including the Associated General Contractors of America (AGC), the Construction Specifications Institute (CSI), and personal contacts available to the research team. The effort resulted in the recruitment of 34 participants between April and May 2020—which represented the data collection period to capture the early effects of the pandemic on the construction industry following the declaration of the national emergency on 13 March 2020. Collectively, the participants possessed over 400 years of experience in the construction industry. Additional information on the background of the participants is presented in Table 1. It needs to be noted that certain participants were part of organizations that focused on multiple construction sectors and were involved in projects across multiple states. Accordingly, the total participant counts in the construction sector category and the state category exceed the total number of participants (i.e., 34) to account for these overlaps. The presented percentage corresponds to the ratio between the count associated with each of the background information categories and the total number of participants (i.e., 34).

The interviews were conducted via telephone to ensure the safety of the research team and the study participants. The responses of the study participants were transcribed as the conversations progressed. Follow-up questions and relevant examples were solicited for each of the questions to enhance the quality of the data and the insights gathered from the effort.

After the interviews were complete, the transcribed qualitative data were imported into the NVivo (Alfasoft, CA, USA) 10 software package for content analysis and coding. NVivo 10 offers powerful features, including the ability to search for keywords and iteratively select codes and subcodes [23]. The interview transcripts were inductively and iteratively coded by the first three authors over multiple meetings until complete consensus was achieved. The codes used corresponded to the three themes that the study targeted, and the subcodes were identified on an evolving and iterative basis as discussed above.

It is important to note that the codes and subcodes adopted were not necessarily mutually exclusive. Rather, the codes and subcodes were adopted to facilitate the presentation of the information gathered in a coherent manner for the purpose of the current article. The findings of the effort are summarized in the following sections.

## 3. Study Findings

**Figure 4:**
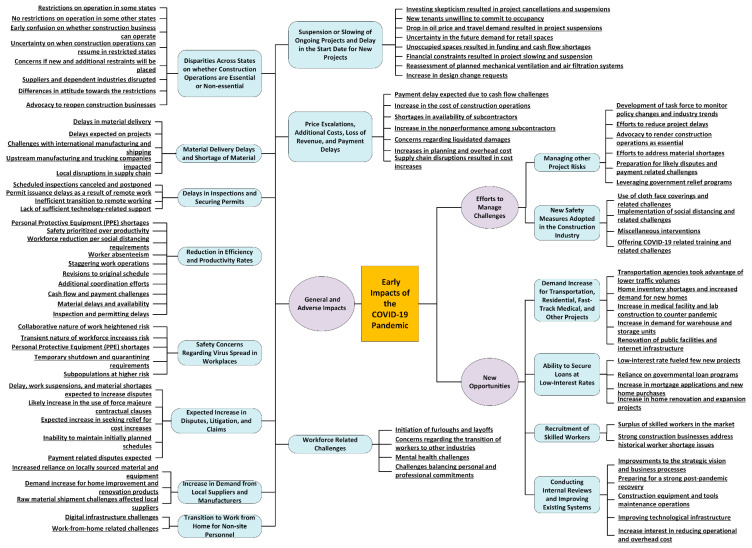
Themes, generated codes, and subcodes from the interviews.

The findings of the effort are organized on the basis of the three themes that the research targeted. However, as mentioned above, there was significant overlap in the content across the targeted areas. Therefore, the purpose of the division of the content in the following sections is only to present the gathered information in a digestible and coherent manner. Overall, the purpose of the organization is to offer a holistic understanding of the early impacts of the COVID-19 pandemic on the construction industry as captured from the interviews. Figure 4 summarizes the generated codes and subcodes under the three main themes (i.e., general and adverse impacts, new opportunities, and efforts to manage challenges).

### 3.1. General and Adverse Impacts of COVID-19 on the Construction Industry

#### 3.1.1. Disparities across States on Whether Construction Operations Are Essential or Non-Essential

In the first few months of the pandemic, stay-at-home or shelter-in-place orders were being enforced across several states to prevent the spread of the novel coronavirus. However, a few states did not require any such restrictions. In most states, construction businesses were deemed to be essential; however, a few states identified construction operations to be at least partly non-essential. Therefore, there were restrictions for construction operations in some states whereas there were no restrictions in other states. Among the states where construction operations were partly restricted, there was much variability in the degree of restrictions. Accordingly, there were many differences in how construction businesses were impacted across different states.

For example, some of the participants mentioned that there was little impact on their projects and that operations continued as normal; although a number of new safety measures were adopted. However, others mentioned that construction operations had completely ceased in their workplaces. A few participants shared that during the first few days, there was significant confusion on whether their business was deemed essential or non-essential due to the variability in the degree of restrictions across states. More specifically, it was unclear to the participants’ employer whether they can continue operations. There was also much uncertainty on when construction operations will resume in several states where restrictions were in place. In other cases, the participants were concerned whether new restraints would be placed if COVID-19 cases increased in their communities.

In cases where construction operations were considered non-essential, one participant mentioned that the impacts are not limited to only construction businesses. The participant shared that upstream suppliers and the operations of several downstream facilities were also adversely impacted. In addition, the participant mentioned that several other industries that serve the construction industry such as manufacturing will also experience negative impacts.

A few of the participants believed that the restrictions were essential given the risk of the virus spread. Others believed that the economic and adverse impacts far exceeded the risk of the virus spread. One of the participants mentioned that several agencies were advocating for the reopening of construction businesses and to change the status of construction businesses as being essential.

#### 3.1.2. Material Delivery Delays and Shortage of Material

Most of the participants reported experiencing or expecting delays in material delivery. These delays in material delivery were also, in turn, expected to delay overall project progress and cause significant schedule disruptions. The delays were particularly relevant when the supply chain involved material or raw material from overseas. For example, one of the project managers mentioned that several building elements were to be shipped from Europe; however, the manufacturing plants were non-operational in Europe due to the COVID-19 pandemic. Others mentioned similar supply chain disruptions involving material and raw material from China, Mexico, Canada, and other nations.

Even within the U.S., although construction was deemed as an essential business in a number of states, several upstream manufacturing units and trucking companies within the supply chain were deemed non-essential. Accordingly, some of these businesses had to halt operations in response to the pandemic. In another case, a vendor mentioned that material delivery delays can also occur because several truck drivers are hesitant to cross state lines due to fears of contracting the virus and the requirement of quarantining for 14 days as imposed by certain states.

Material shortage was also experienced as a result of the social distancing and quarantining requirements that resulted in a smaller workforce within supply chain organizations. In many cases, the study participants were unable to predict the amount of delays given the number of factors that can impact delivery time in a pandemic situation.

#### 3.1.3. Delays in Inspections and Securing Permits

Significant delays in inspections and securing permits were also reported. For example, one of the interviewed contractors mentioned that as all parties transitioned to the new format of working, there were delays in completing inspections and the certification of work. More specifically, the contractor mentioned that the owner’s representative in many cases was unable to stick to the initial timeframe to complete the certification of completed work. In many cases, several inspection-related meetings had to be canceled and postponed due to the challenges and restrictions of meeting in person.

The participants also shared that there were delays with securing permits from various governmental agencies. These delays were largely due to governmental agencies transitioning to working remotely from home and challenges associated with accessing the necessary information and documentation. In fact, in many cases, designers, architects, and project engineers mentioned that governmental agencies did not have an efficient and working system in place to make such a rapid transition. There were also discussions on the lack of sufficient technology-related support for these agencies as they grappled with making operational changes to the permitting process. In a few cases, the participants mentioned that the permitting processes were suspended temporarily until the governmental agencies were able to set up an online protocol for issuing the necessary permits.

#### 3.1.4. Reduction in Efficiency and Productivity Rates

Productivity rates reportedly suffered across the construction industry. Much of the loss in productivity and efficiency was attributed to the new safety measures that were necessary to protect the workforce as the pandemic continued to progress. In fact, one of the project managers indicated that working safely was the top priority, and productivity took a backseat in the pandemic situation. Reduction in productivity rates also was attributed to shortages in the availability of PPE and the reduction in the number of workers to comply with the social distancing recommendations. In many cases, the participants also mentioned that workers choose to not report to work for a variety of reasons, which also impacted productivity and efficiency. Some of the reasons mentioned included quarantining requirements, caring for children as a result of school closure, and the fear of being infected at work and being carriers of the virus when around family. Recruitment and training of replacement workers also consumed substantial amounts of time.

The staggering of subcontractors in such a way so that they do not work alongside other subcontractors also was mentioned as affecting productivity rates. One of the participants mentioned that such staggering required significant revisions to the initially planned schedule and required much additional work and coordination. Inefficiencies in coordination along with the safety contingencies that were applied yielded lower productivity levels.

Cash flow challenges experienced by contractors and subcontractors were also mentioned as affecting productivity. Cash flow issues were particularly a problem due to escalating material prices and challenges that owners experienced with making timely payments to the contractors. Finally, as already discussed above, delays in material delivery, shortages in material availability, and delays in inspections and permitting were all also associated with productivity losses.

#### 3.1.5. Suspension or Slowing of Ongoing Projects and Delay in the Start Date for New Projects

Given that the pandemic caused widespread economic downturns and uncertainties, owners, investors, and businesses were increasingly wary about investing in construction projects and operations. Therefore, a number of projects were canceled or temporally suspended. For example, one of the participants mentioned that several developers of commercial property, in particular, are increasingly waiting on the commitment from potential tenants before they can begin customized construction operations. However, the volume of potential tenants willing to make a commitment has significantly reduced as a result of the pandemic. In the same manner, one participant mentioned that with the plunge in the price of oil and the drop in the travel demand, a number of projects in the oil and gas sector have been suspended. The participant also mentioned that the budget devoted to oil and gas projects in the near future is expected to dramatically reduce.

In many other cases, the study participants reported that several private owners are citing financial concerns with the broader market and requesting that construction operations be either slowed or stalled. According to one architect, with the increasing number of individuals working, shopping, and studying over the internet, the future of retail and commercial property remains uncertain. The participant also mentioned that it is unlikely for the demand in these sectors to rebound in the immediate future, and hence, it may not be financially prudent to make progress in many of the ongoing projects.

One of the owners asked the contractors to slow the completion of a student apartment since students were not expected to report to the college town as classes were being taught online and cash flow was tight. Several of the owners experienced cash flow issues from existing properties and were unable to fund the completion of additional properties.

New construction projects were particularly impacted. One project engineer working for an industrial contractor reported that nearly 90% of the projects that were in the front-end loading (FEL) phase (i.e., pre-project planning) were put on hold. Moreover, several projects that were in the bidding stage were also canceled or postponed. One of the participants mentioned that fewer projects will be approved and funded as a result of the pandemic compared to previous years.

Another important reason for slowing down ongoing projects was the increase in requests to reassess mechanical ventilation and air filtration systems. One of the architects mentioned that owners were now concerned with whether their mechanical ventilation and air filtration systems would adequately replace contaminated air with clean air. In several cases, owners requested upgrades to the initial design to offer superior occupancy safety. There were also concerns on whether design changes will need to be incorporated in a post-pandemic world in anticipation of future events.

#### 3.1.6. Price Escalations, Additional Costs, Loss of Revenue, and Payment Delays

According to several of the participants, supply chain disruptions resulted in an increase in the cost of construction materials. As discussed earlier, much of the disruption resulted from the closure and reduction in the capacity of manufacturing and processing facilities that are upstream in the supply chain. The increase in the cost of lumber, cement, and concrete products was particularly highlighted by a number of participants. Along with the increase in the cost of material, an increase in the cost of doing business was also reported. In many cases, the participants mentioned that this resulted in unexpected revenue and financial shocks at various points in the supply chain. In some cases, the participants mentioned that construction businesses may have to handle the extra costs themselves unless there is relief from the owners and other stakeholders as per the contractual agreement. This included the additional costs of managing safety, offering pandemic-related safety training, and securing the necessary PPE to sufficiently protect the workforce.

An increase in costs was also experienced since fewer subcontractors were willing to work and travel during the pandemic. According to one of the project managers, subcontractors that had to cross state lines were particularly hesitant to work given that they preferred to stay in their city of residence and avoid the 14-day quarantining requirement imposed by some states. In such cases, subcontractors often had to be offered larger compensations and incentives, which resulted in higher costs and potentially lower quality. An increase in the number of non-performance occurrences among subcontractors was also reported to increase costs.

Several contractors also expressed concerns with respect to additional costs from liquidated damages. The contractor representatives mentioned that the delays caused in the projects can result in these additional liabilities, and this can, in turn, impact the profit margins and the success of the projects.

Contractors were concerned that their staff will spend additional time on projects as a result of the experienced delays. This additional time was expected to translate into additional costs and overhead. Likewise, owners were concerned about the loss of revenue that was expected due to project delays and unoccupied retail and commercial properties. The participants were also concerned regarding the additional costs associated with the planning efforts that were necessary to transition successfully through the pandemic. Others alluded to the cost of shutting down projects and restarting the project as a major cost item that construction businesses will largely have to bear.

As construction operations are delayed, several participants mentioned that payment delays are likely to follow. The participants believed that this can result in cascading cash flow issues, and some contractors may struggle to pay their workforce, subcontractors, and suppliers in a timely manner.

#### 3.1.7. Safety Concerns Regarding Virus Spread in Workplaces

There was much concern regarding the spread of the virus in construction workplaces. The participants were of the opinion that construction work is inherently collaborative and requires that different trades work alongside each other. In many cases, this requires workers to share workspaces and facilities, including portable bathrooms. Therefore, some of the participants believed that the risk of virus spread is significantly high and that robust safety measures were to be adopted to protect workers. However, given the collaborative nature of the work, several participants mentioned that safety guidelines such as social distancing were not very feasible in construction workplaces. In fact, concerns were expressed that the safety measures recommended for adoption in construction workplaces may have been developed without any consultation with construction professionals that are more familiar with construction operations. Additional information on these challenges is presented in greater detail in Section 3.3.1.

Some of the participants mentioned that the risk of transmission is particularly high given that infected individuals may not particularly experience any symptoms in the early stages of the infection and can be active carriers of the virus. There was also mention that the risk was exacerbated by the fact that tests were not widely available, and even when workers had access to tests, there was usually a delay in receiving the test results. One of the supervisors mentioned that the risk of spread is undoubtedly higher in industries that are identified as essential but experience shortages in PPE and lack effective measures to prevent the virus spread.

Others discussed that several subcontractors work on different sites. Therefore, one of the participants mentioned that these subcontractors can spread the virus from one workplace to the other. In fact, according to one of the project managers, one of the subcontractor employees that newly transitioned into a workplace tested positive for the virus. In response, the project had to be temporarily shut down and the other workers that worked in the proximity of the positively tested worker had to quarantine for 14 days. This resulted in significant paperwork and related challenges. Similar challenges were expected as a result of the transient nature of the workforce including independent contractors. Suppliers and delivery personnel that visit different sites were also expected to pose a higher risk of virus spread.

A few participants shared that Hispanic and Latino workers may particularly be at a heightened risk of infection given their greater involvement in essential work within and outside of the construction industry. Others mentioned that the lower wages, immigration status and associated challenges, social connections with other essential workers, and their role as independent contractors in the industry expose these workers to higher risks.

#### 3.1.8. Expected Increase in Disputes, Litigation, and Claims

Most of the participants expected that there will be a significant increase in the number of disputes, litigations, and claims on their projects and across the industry. They believed that the delays, temporary suspension of work, material shortages, and the additional costs that resulted from the pandemic will likely be the underlying cause for the disputes, litigations, and claims. The majority of the conflicts were expected between contractors and owners for nonperformance and delays. However, disputes were also expected in contractor-subcontractor, contractor-supplier, owner-future tenant, and contractor-insurance entity relationships.

Given the circumstances, the participants mentioned that there will be an increase in the use of the force majeure contractual clause, where contractors and subcontractors claim that the delays experienced were caused by circumstances that were unforeseeable and beyond their control. If contractors and subcontractors are able to successfully offer the relevant evidence, they may be able to secure a time extension and relief from delay-related penalties. In circumstances where a force majeure or another relevant contractual clause is absent in the contractual agreements, the contractors may seek relief in other ways. Disputes and conflicts become particularly likely when there are disagreements between the parties about the cause of the delay or the interpretation of the force majeure-related contractual language. The participants also mentioned that likelihood of disputes and litigation can increase if the owner has a future tenant that has committed to occupying the constructed facility.

Some participants also mentioned that contractors and subcontractors may seek relief for the increase in cost. The likelihood of success in these circumstances is dependent on the pricing mechanism adopted as per the contractual agreement. Contractors and subcontractors will also likely examine the contract to see if there are provisions to claim additional compensation using price escalation clauses. In some situations, there could also be disagreements with insurance agencies on whether the insurance policies offer coverage for a broad array of expected construction risks, including non-performance.

Other areas for conflict that the participants shared included the inability of suppliers to deliver construction materials in a timely manner, suppliers seeking to cancel delivery commitments, and suppliers seeking additional compensation for losses sustained from supply disruptions.

There were also many discussions about disputes related to payments. Because of the market uncertainty, cost overruns, and cash flow challenges, it was expected that owners, contractors, and subcontractors will experience financial hardships. Therefore, the parties involved may struggle with making timely payments and honoring the commitments if cash reserves are unavailable to them. In the context of contractors and subcontractors, one of the participants mentioned that the biggest challenge is going to be cash flow issues. More specifically, the participant shared that contractors and subcontractors will need to pay suppliers after the material is delivered; however, they will receive their payment only once the material is incorporated in the facility under construction. Therefore, contractors and subcontractors were particularly expected to experience cash flow-related challenges and claims.

Regardless of the conflicts and disputes experienced in the current pandemic, several participants mentioned that the pandemic has prompted industry stakeholders to take a closer look at the contractual documents and examine clauses that offer relief in uncertain circumstances. Additional information on some of the risk management efforts planned in the industry is presented in Section 3.3.2.

#### 3.1.9. Workforce-Related Challenges

The participants shared considerable information about workforce-related challenges. Most participants shared that a large number of furloughs and layoffs are being initiated as the workloads in the construction industry are projected to decrease substantially. In fact, one of the project engineers mentioned that the projected workload reduction will range between 50% and 60% compared to the previous years. In addition, several participants mentioned that contractors and subcontractors are experiencing significant challenges with cash flow and that these furloughs and layoffs are necessary, as these businesses will not be able to make the payroll. Moreover, several participants mentioned that there is much uncertainty in the future and that the demand for craft workers remains unclear given the early stages of the pandemic. Apart from craft workers, project engineers, estimators, administrative employees, and others were also considered likely to be impacted by furloughs and layoffs.

A significant challenge reported by some of the participants was the long-term effects of the pandemic on the construction workforce. For example, several project managers and supervisors alluded to the economic downturn that was previously experienced during the Great Recession when a large number of construction workers abandoned the construction industry to join other industries. These participants mentioned that these workers never returned to the construction industry following the recovery and that this has resulted in a large deficit in the number of skilled workers in the industry. The participants were worried that the current pandemic will further aggravate the situation if the laid-off workers choose to transition to other industries and not return.

Another problem that was discussed was the mental health challenges that workers experience in these uncertain times. For example, a few participants mentioned that a significant portion of the workforce are worried about their future prospects in the industry and the possibility that they may be laid off or furloughed. One participant mentioned that these workers have their own financial obligation, a family to care for, and rent and housing payments to make—which are all significant stressors for the workers. Some others, as discussed earlier, mentioned that workers are anxious that they may be exposed to the virus in the workplace and that many of the safety measures are not realistic to adopt or are not sufficiently enforced in construction workplaces. In addition, as discussed earlier, a supervisor mentioned that workers are often unable to report to work due to childcare unavailability, closure of schools, and caretaking responsibilities for sick family members, and these stressors impact their mental health.

One of the project managers mentioned that the social distancing requirement is also resulting in limited interactions among crewmembers, which may also contribute to mental health challenges. The project manager added that he had come across the term physical distancing, which must replace the use of the term social distancing. The participant believed that such a change would better communicate the intent of the social distancing safety measure and argued that complementary efforts must be adopted to build comradeship between workers during this difficult time.

#### 3.1.10. Increase in Demand from Local Suppliers and Manufacturers

Given that there were significant delays with material delivery, particularly from overseas and across the country, many workplaces began to proactively adopt measures to find alternative material sources to reduce the risk of project delays. Preference was given to alternate local suppliers and manufacturers where the likelihood of delivery on short notice was higher. In many cases, contractors, in consultation with the architects and the designers, were able to identify alternate material and equipment that local suppliers and manufacturers were able to quickly ship. Therefore, these local suppliers experienced a significant spike in demand. More specifically, according to one of the suppliers, their sales substantially increased when compared to previous years. There was also additional demand from local suppliers for supplies such as disinfecting wipes, cleaning supplies, sanitizers and sanitizer stations, and acrylic glass (e.g., Plexiglas) that were necessary as safety measures during the pandemic for offices, retail spaces, and educational institutes.

In addition, because of the stay-at-home orders, a supplier mentioned that a larger proportion of the public began new home improvement and renovation projects. Therefore, there was an increase in demand for material for these small-scale projects. However, the supplier also mentioned that they were running low on stocks since they had not anticipated or planned for a surge in the demand. Moreover, these local suppliers were also unable to receive shipments of the raw material that they needed from outside sources to meet the local demand.

#### 3.1.11. Transition to Work from Home for Non-Site Personnel

In response to the pandemic and to enhance safety, a significant number of non-site professionals were able to transition to working from home. However, several participants mentioned that they experienced a significant number of challenges. For example, businesses did not have the necessary digital infrastructure to make the transition easy in many cases. Therefore, there were significant challenges with gaining access to necessary software packages and other resources, which resulted in much inefficiency. In many cases, the businesses needed to make additional investments into technology to enhance their ability to efficiently work from home. For example, several businesses invested in virtual private networks (VPNs) to gain access to resources and software packages such as computer-aided design (CAD) remotely. Few organizations mentioned that they already had cloud solutions to access licensed software packages and business databases—which made the transition relatively easy.

In several other cases, the participants mentioned that there were additional challenges with making the transition because much of the workforce was not familiar with the newly adopted digital solutions. For example, certain individuals were not familiar with using virtual private networks (VPNs) to connect remotely to the business network. Others experienced challenges with adopting new communication platforms such as Zoom, Microsoft Teams, and Slack. Some employees also found it challenging to connect their business computers to their home network for a variety of reasons. Few participants also mentioned Internet outages and poor Internet quality as major challenges associated with remote working.

Apart from technology-related challenges, a few participants mentioned that there were more distractions at home that interfered with their ability to work effectively. In addition, many of the employees also had additional duties at home such as taking care of their children, given that schools were online and childcare services were not operational.

Because of the many challenges that employees experienced with technology and working from home, a few organizations decided to ask their employees to return to the office after providing safety measures such as acrylic (e.g., Plexiglas) panels between desks, sanitation stations, and sufficient spacing between workspaces.

### 3.2. New Opportunities as a Result of the COVID-19 Pandemic

#### 3.2.1. Ability to Secure Loans at Low Interest Rates

The participants shared that one of the largest opportunities comes from the low interest rates that are available to owners, contractors, and potential home buyers. For example, one of the project managers indicated that they just secured a new project involving the construction of a hotel as the owner was able to secure a loan with a much lower interest rate than usual. The project manager also mentioned that such low interest rates are rare and that this offers a good opportunity for strong businesses to grow and be prepared to take advantage of the markets when the recovery begins. There were also participants that discussed the availability of the small business loan program, commonly known as PPP loans—the Paycheck Protection Program (PPP)—that small contractors were leveraging to ensure that their employers were paid and that their workforce successfully weathered the ongoing pandemic. According to the U.S. Small Business Administration (SBA), the first draw of PPP loans had an interest rate of 1% [24].

The participants also mentioned that the demand for new residential construction is likely to increase because potential buyers can secure a low interest rate for the life of the mortgage, which can go up to 30 years. One of the participants mentioned that a few families are expected to spend more on residential expansion projects by leveraging the low interest rates as they will spend more time in their homes, which will benefit small contractors. Furthermore, a few participants mentioned that potential home buyers are moving from rented properties in cities to the suburbs, where they can purchase larger and more spacious homes using the low interest rates; which will also increase sales in the residential sector.

#### 3.2.2. Demand Increase for Transportation, Residential, Fast-Track Medical, and Other Projects

A large number of participants shared that the demand for a variety of construction projects has increased. For example, one of the project managers stated that Departments of Transportation (DOTs) are approving new projects and accelerating ongoing ones to take advantage of the lower traffic volumes following the shelter-in-place orders. The project manager added that this is a rare opportunity to work on infrastructure and highway projects, particularly in urban areas, where high traffic is typically a major concern and can result in much inconvenience and delays to drivers. In fact, one of the contractors from Texas reported that the limited volume of traffic has offered more lane closure opportunities and has allowed for the acceleration of projects. Another project engineer reported that the pandemic has enhanced the safety and productivity of workers as the traffic flow has reduced. Another manager mentioned that the pandemic has offered an opportunity to safely work on infrastructure projects that have been neglected for decades.

In terms of residential projects, apart from the additional demand following the reduction in mortgage and interest rates, demand for new construction was expected to increase as the inventory of homes in the market was expected to reduce. This was because fewer owners of existing homes were expected to list their homes for sale and allow public access to their properties during a pandemic. The shortage of inventory was expected to increase the sale of new homes.

Apart from transportation and residential projects, given the medical emergency that was being experienced due to the pandemic, the participants believed that more fast-track medical and patient care facilities will be built and renovated. Additional construction projects were also expected for building new research centers and medical labs to counter future medical emergencies.

In the commercial sector, many participants believed that there will be an increase in the construction of warehouses and storage units to support the substantial increase in online shopping. The participants mentioned that many retail spaces are also likely to be renovated as new warehouses as the demand for retail space reduces. The demand was further expected to increase as a growing number of businesses were expected to transition into the online shopping space.

Other projects that were expected to increase as a result of the pandemic included renovation work in universities and schools as students were away from campus, office renovations in preparation for employees to return and work safely during a pandemic, utility work as the number of Internet subscribers increased and traffic flow reduced, and renovation of public buildings such as museums and public offices with the decline in the number of visitors.

#### 3.2.3. Recruitment of Skilled Workers

As discussed above, a large number of furloughs and layoffs were expected, not only from the construction industry but also from other industries including retail, manufacturing, and airlines. While this was an adverse outcome for many organizations and workers, a few participants believed that their organizations benefitted from the situation. More specifically, these participants stated that while the industry in the past suffered from a shortage of skilled workers, suddenly, there was a surplus of skilled workers looking for positions in the market. A project manager mentioned that their organization was able to recruit a number of skilled and experienced electricians that were typically difficult to come across in the industry. The project manager mentioned that well-established and financially strong contractors will particularly benefit from their ability to hire a strong workforce.

#### 3.2.4. Conducting Internal Reviews and Improving Existing Systems

With the reduction in the workload experienced in the construction industry and fewer new opportunities, many construction businesses resolved to perform comprehensive evaluations of their processes and systems to identify areas for improvement. According to several participants, their construction businesses began to examine their strategic vision, partnerships, their execution plans, their bidding approach, risk assessment approach, material planning protocol, software resources, and others to identify inefficiencies and strategic efforts that can be adopted to enhance success. According to one participant, these businesses focused on coming out stronger at the end of the pandemic and ready to thrive in a post-pandemic market with improved processes and systems.

Several organizations focused on performing maintenance operations for their construction equipment and tools. Two project managers mentioned that their organizations are examining the prospect of switching a larger number of positions to work-from-home positions. These managers argued that such a change can allow them to transition to a smaller office while reducing overhead costs and improving flexibility. Several businesses resolved to improve their technological infrastructure to better facilitate collaborations between suppliers, subcontractors, and other partners. One of the participants shared that their organization was in the last stages of proposing a new organizational structure to enhance communication channels and accountability.

### 3.3. Efforts to Manage the Challenges of the COVID-19 Pandemic

#### 3.3.1. New Safety Measures Adopted in the Construction Industry

The participants reported a number of safety measures that were being adopted, along with their implementation challenges. Among others, the use of cloth face coverings was enforced widely in construction workplaces. However, in many cases, there were significant shortages in the availability of face coverings in the market and workplaces. Moreover, workers often forgot to bring their face coverings to work when they returned the next day, which further aggravated the shortage of face masks and coverings in many cases.

Even when face coverings were available, several supervisors reported that it was challenging to enforce the requirements in workplaces. For example, several workers often removed the face coverings due to discomfort and fogging of safety glasses. Workers also often complained of their discomfort with the masks, especially when performing strenuous tasks that were associated with a higher breathing rate and perspiration. Workers also expressed discomfort when working in humid conditions with their face coverings. In many other cases, workers often covered their mouths but did not cover their noses, which limits the effectiveness of cloth face coverings. Another supervisor mentioned that even when workers placed the mask correctly over their nose and the mouth, it often would not stay in place and would fall below the nose as they worked. Another significant challenge was that workers often reused face coverings without rewashing them. However, this was unrealistic to monitor according to one of the supervisors.

Workers and employees were also given instructions to follow social distancing guidelines. In addition to promoting social distancing efforts, several other approaches were adopted to support social distancing among site personnel. These included staggering work crews such that different work crews reported to work at different times to limit the number of workers in the workplace at the same time, posting signage to remind workers of the requirements of social distancing, preventing food trucks from coming into the site that typically encouraged crowding, limiting the number of workers in the breakroom, setting up open public break and lunch spaces, closing the site trailer, use of social distancing floor markers placed 6 ft. apart, encouraging non-essential workers to work from home, asking supervisors to work from their trucks or vehicles, reducing and postponing work in confined spaces, and others. While these efforts were all useful, some participants mentioned that it was unrealistic to maintain the 6 ft. requirement between each of the workers at all times due to the collaborative nature of construction operations, where workers are required to work alongside each other. Moreover, like monitoring the usage of face covering, it was unrealistic for site personnel to monitor that workers always followed social distancing guidelines as work progressed.

One of the supervisors mentioned that when workers used face coverings, many believed that the 6 ft. social distancing requirement no longer applied, as the face covering offered sufficient protection. The supervisor mentioned that this belief is untrue and the recommended practice is to adopt multiple safety measures to limit the likelihood of virus spread. In a number of instances, the supervisor mentioned that safety professionals must intervene were when such behaviors are observed.

Another widely adopted safety measure was offering workers and site personnel training on the safety risks of COVID-19 and the safety measures that are to be adopted. The biggest challenge of delivering these training sessions was that the traditional classroom-type training approaches were no longer safe given the social distancing guidelines. Therefore, new training approaches that comply with the social distancing requirements were necessary. Some of the employers tried to adopt online training sessions. However, technology constraints were a significant issue because workers often did not have access to compatible computers and phones. Computer literacy and access to a good Internet connection were also a challenge in many cases. In several other cases, the contractors and the subcontractors did not have the infrastructure necessary to deliver training programs online given the unexpected demand for online training. Therefore, in some cases, training was offered over conference phone calls. However, trainers struggled with this format of training since the conversations were largely one-sided, with little to no participation from the training participants. In many cases, according to a participant, it was not clear if the workers were actually paying attention to or benefiting from the training. Moreover, the inability to see the facial expressions of the training participants was a challenge for trainers as they were unable to see the facial expressions as would be the case in a classroom-type training setting. To overcome all of these challenges, some of the employers offered the training in open spaces in the workplace while also complying with the social distancing requirements.

Other safety measures that were adopted without much challenge were administering temperature checks prior to entry into the workplace, the placement of sanitizers and hand wash stations at the entrance and various locations in the worksite, disinfecting tools and surfaces, discouraging sharing of tools and equipment, including PPE, encouraging workers with any COVID-19-like symptoms to remain at home, adopting air purification and filtration systems, and others. One concern that was reported by a supervisor regarding the temperature checks was the concern regarding the accuracy of these checks. More specifically, the supervisor was concerned that the temperature checks may be impacted by ambient temperature and environmental conditions if testing was performed in a non-controlled location (e.g., open space at the entrance to the workplace).

#### 3.3.2. Managing Other Project Risks

Given the rapid change in governmental policies during the pandemic, several participants reported that their businesses maintained a task force or team of qualified employees or consultants who were tasked with examining proposed governmental policies and industry patterns to identify best practices for risk management. In many cases, such a task force or team was also delegated the responsibility of identifying and adopting safety measures that were recommended for the safe operation of construction workplaces. The taskforce continuously updated operational plans and offered guidance to the site leadership on managing work in construction workplaces. In one of the projects, while they did not have the resources to support a task force, one of the project managers examined the lessons learned database and past claims to identify possible solutions that were proposed in the past that may apply in the current crisis.

Much of the input that participants shared was related to managing delays on their projects, which appeared to be the largest risk apart from the safety concerns being experienced. Some participants mentioned that delays on their projects were inevitable and only the impacts of the delay can be minimized. Others mentioned that they were reviewing their original schedules to make modifications that may be helpful in reducing delays. For example, some participants mentioned that they were considering the adoption of night and weekend shifts. One of the project managers mentioned that the introduction of additional shifts would also be helpful given that one of their subcontractors was not willing to share the workplace with other trades because of the safety risks during the pandemic. Several other participants mentioned the work of several advocacy groups, trade unions, and associations such as the Associated General Contractors (AGC) of America, North America’s Building Trades Unions (NABTU), and the American Subcontractors Association (ASA) that were urging governmental agencies to render construction activities as being essential for recovery and offering exemption from shelter-in-place orders. According to these participants, if these efforts are successful, significant delays and associated hardships will be alleviated.

Another area that was highlighted was efforts to reduce the impacts of material delivery delays and related challenges. One of the participants mentioned that some businesses that were proactively monitoring the evolving pandemic were able to place material delivery requests prior to when deliveries became more challenging and shortages of supplies were experienced in the market. Some other subcontractors were able to resolve these issues by finding alternate vendors and local suppliers that were able to deliver material on time. Several other contractors were examining their project schedule to identify material that was needed throughout the project cycle and actively contacted vendors to ensure that they will be able to make deliveries as planned. Nonetheless, several workplaces were unable to find alternate approaches to have the materials delivered as per initial plans—particularly when customized products and fabricated elements were necessary.

As projects were experiencing delays and price escalations, several participants mentioned that they began reviewing the contracts to assess expected impacts and provisions that they can use in their defense. Several participants mentioned that they were keeping detailed records of the causes of the delays and price increases with the hope of requesting additional time and compensation. Such records were also expected to be useful to manage the risk of liquidated damages and delay-related penalties. Several of the participants also mentioned that such records will be useful for their defense if disputes or ligations occur on their projects. Apart from these efforts, few project managers mentioned that they were working on notices that were planned to be sent to project owners, alerting them of expected delays and cost escalations.

One of the other major risks that was expected was cash flow issues as a result of price escalations and delayed payments. Several participants mentioned that businesses were taking advantage of various governmental programs and relief plans to alleviate some of the challenges. These included new loans and economic relief plans that were becoming available for small businesses such as the PPP. One of the supervisors mentioned that an employer planned to use these programs to pay their workforce and ensure that they maintain their trained employees and skilled workers despite the pandemic. The supervisor mentioned that this was important for the long-term success of the business, as the construction industry has struggled with labor shortage issues. One of the participants mentioned that their organization maintained a generous contingency and emergency account which will be used to address the experienced cash flow challenges.

## 4. Study Limitations and Suggested Future Efforts

While the study makes important contributions, there are a few limitations that may be addressed in future efforts. First, although the study captures important findings related to the effects of the COVID-19 pandemic on the construction industry, there may be additional effects that may not have been captured in the current study due to a number of reasons. For example, the exploratory nature of the study was designed to only capture the effects of the pandemic as experienced by the study participants—who may not sufficiently represent the industry as a whole. While the participants represented 17 states across four different sectors with varying roles, there are a number of states and stakeholders that were not represented in the reported effort due to the time constraints associated with capturing the early effects of the pandemic and limitations on the resources available to the research team. Nonetheless, it needs to be noted that the participating professionals were socially connected with other professionals and stakeholders across the U.S. and shared a significant amount of pertinent and useful information.

Second, the scope of the article was limited to the early effects of the pandemic as experienced between the declaration of the national emergency in the U.S. on 13 March 2020 and 30 May 2020, when the data collection efforts were concluded. Therefore, the effects of the pandemic and the related management efforts that may have occurred after the targeted timeline may vary. For example, given the evolving nature of the pandemic, it is unclear what the future will look like as the industry continues to grapple with the pandemic.

Third, while the current effort offers an overview of the effect of the pandemic on the construction industry as reported by industry stakeholders, it does not offer quantitative findings such as the average delay experienced, the average cost overruns expected, or the financial impacts on organizations. The study was also not designed to capture the prevalence of the reported challenges in the construction industry given the limited number of study participants. Future efforts may build upon the current findings to quantify the impacts of the pandemic on the industry. Future effects may also focus on investigating the effects of the pandemic on the global construction industry and developing universal state-of-the-art solutions to tackle the continued crisis. Efforts may also focus on vetting interventions and best practices to prepare for future unexpected pandemics and emergencies.

## 5. Conclusions

The COVID-19 pandemic has resulted in substantial disruptions and hardships across nations and industries. Like other industries such as airlines, retail, and restaurants, the construction industry has also been impacted in a number of ways. Through interviews with SMEs, the current article focused on cataloging the early impacts of the pandemic as reported by construction stakeholders. The study findings identified that the construction industry experienced a number of adverse effects. These included material delivery delays, shortage of material, permitting delays, lower productivity rates, cash flow-related challenges, project suspension, price escalations, and potential conflicts and disputes.

Despite the number of challenges, there were a number of new opportunities that were experienced in the construction industry as a result of the pandemic. These included opportunities that resulted from lower interest rates; demand increase in the medical, transportation, and residential sectors; and the ability to recruit skilled workers.

The research effort also unveiled specific efforts that were adopted to manage the challenge of the COVID-19 pandemic in construction workplaces. These included safety measures such as requiring workers to wear face coverings, implementing social distancing guidelines, adopting COVID-19-related safety training, and encouraging work-from-home initiatives. Other risk management measures to combat the effects of the pandemic included establishing a task force that is tasked with offering COVID-19-related guidelines, proactive steps to reduce the risk of delays, advocacy efforts seeking to establish construction operations as being essential, and leveraging governmental relief programs to preserve businesses and the workforce.

The presented research offers an understanding of the impacts of the COVID-19 pandemic on the construction industry. The findings of the effort will be useful to governmental agencies as they seek to elevate the adverse effects experienced in the construction industry. Industry representatives may use the findings to identify risk management efforts that may be appropriate for their own organizations. Researchers may use the findings to identify problem areas and propose relevant interventions to support the efforts of the industry.

## Figures and Tables

**Table 1:** Summary of the participants and their organizations’ background information.

Construction Sector		
**Sector**	**Count**	**Percentage (%)**
Commercial	23	67.65
Industrial	6	17.65
Infrastructure	9	26.47
Residential	11	32.35
**Organization Type**		
**Type**	**Count**	**Percentage (%)**
Contractor	25	73.53
Owner	7	20.59
Supplier	2	5.88
**Job Role**		
**Role**	**Count**	**Percentage (%)**
Project Manager/Engineer	26	76.47
Architect/Designer	6	17.65
Superintendent	2	5.88
**Organization Size**		
**Size**	**Count**	**Percentage (%)**
Less than 100	14	41.18
100–500	9	26.47
More than 500	11	32.35
**Project Location (State)**		
**State**	**Count**	**Percentage (%)**
Florida	9	26.47
Texas	5	14.70
Virginia	4	11.74
North Carolina	4	11.74
California	3	8.82
Arizona	2	5.88
New York	2	5.88
Illinois	1	2.94
South Carolina	1	2.94
Georgia	1	2.94
Kansas	1	2.94
Pennsylvania	1	2.94
Indiana	1	2.94
Washington	1	2.94
Arkansas	1	2.94
Washington, D.C.	1	2.94
New Jersey	1	2.94

## Data Availability

The data are available upon request.
